# Piperlongumine Induces Cell Cycle Arrest via Reactive Oxygen Species Accumulation and IKKβ Suppression in Human Breast Cancer Cells

**DOI:** 10.3390/antiox8110553

**Published:** 2019-11-14

**Authors:** Chang Hee Jeong, Haram Ryu, Do Hyun Kim, Wei Nee Cheng, Jee Eun Yoon, Sukyung Kang, Sung Gu Han

**Affiliations:** Department of Food Science and Biotechnology of Animal Resources, Konkuk University, Seoul 05029, Korea; hello01@konkuk.ac.kr (C.H.J.); gkfka1005@naver.com (H.R.); tyche311@naver.com (D.H.K.); herm_es@hotmail.com (W.N.C.); 7wlmds7@naver.com (J.E.Y.); sukyungkang@greencross.com (S.K.)

**Keywords:** piperlongumine, cell cycle arrest, oxidative stress, glutathione depletion, IKK suppression, reactive oxygen species (ROS) accumulation

## Abstract

Piperlongumine (PL), a natural product derived from long pepper (Piper longum L.), is known to exhibit anticancer effects. However, the effect of PL on cell cycle-regulatory proteins in estrogen receptor (ER)-positive breast cancer cells is unclear. Therefore, we investigated whether PL can modulate the growth of ER-positive breast cancer cell line, MCF-7. We found that PL decreased MCF-7 cell proliferation and migration. Flow cytometric analysis demonstrated that PL induced G2/M phase cell cycle arrest. Moreover, PL significantly modulated the mRNA levels of cyclins B1 and D1, cyclin-dependent kinases 1, 4, and 6, and proliferating cell nuclear antigen. PL induced intracellular reactive oxygen species (hydrogen peroxide) accumulation and glutathione depletion. PL-mediated inhibition of IKKβ expression decreased nuclear translocation of NF-κB p65. Furthermore, PL significantly increased p21 mRNA levels. In conclusion, our data suggest that PL exerts anticancer effects in ER-positive breast cancer cells by inhibiting cell proliferation and migration via ROS accumulation and IKKβ suppression.

## 1. Introduction

Breast cancer is the most frequently diagnosed type of cancer among women. According to a recent report from the World Health Organization (WHO), approximately 0.6 million women died of breast cancer worldwide in 2018, which accounts for 15% of the total cancer-related deaths among women [1]. Most of the cell growth in breast cancer is mediated by estrogen and the estrogen receptor (ER, e.g., ERα and ERβ) [2]. Upon activation by estrogen, ER can deliver signals into the nucleus via regulation of transcription factors, including activator protein-1 (AP-1) and nuclear factor-κB (NF-κB) [3,4]. Approximately two-thirds of the breast cancers are ER-positive; in these cancers, cell growth and proliferation are dependent on the presence of estrogen [5]. Therefore, a breast cancer cell line, MCF-7, which is ER-positive, was employed in this study.

The preventive and therapeutic effects of Piperaceae against cancers are well recognized. Especially, piperlongumine (PL), a natural alkaloid extracted from long pepper (Piper longum L.) [6], is known to exert selective anticancer effects in breast, stomach, and lung cancers through the induction of apoptotic cell death [7,8,9]. Accumulation of reactive oxygen species (ROS) in cancer cells due to PL treatment interferes with the intracellular redox mechanisms [10]. For example, PL directly interacts with Keap1, which leads to Nrf2 activation and upregulates HO-1 expression, thereby resulting in the selective killing of cancer cells in the breast [9]. In addition, PL induced apoptosis of breast cancer cells via activation of transcription 3 (STAT3) and phosphatidylinositol 3-kinase (PI3K)/Akt/mammalian target of the rapamycin (mTOR) signaling pathway [11,12]. Although previous studies have indicated that the anticancer effects of PL are associated with cell cycle arrest and apoptosis in many cell types, there are limited studies related to cell cycle-regulatory proteins and the underlying cellular mechanisms in breast cancer cell lines, including MCF-7.

Accelerated cell cycle progression is one of the main mechanisms underlying cancer development that leads to uncontrolled cell proliferation and migration [13]. Cell cycle progression is controlled by the sequential activation of cyclin-dependent kinases (CDKs) and their corresponding cyclins [14]. The generation of ROS is also associated with the regulation of cell cycles. Indeed, data from multiple studies have demonstrated that intracellular ROS can arrest cells in the G0/G1 phase and induce apoptosis through the caspase pathway [15,16]. In addition, ROS accumulation was reported to inhibited NF- κB activation by decreasing IKK expression [17]. Since NF-κB promotes cell cycle progression, the inhibition of NF-κB activation can lead to cell cycle arrest and a decrease of cell proliferation [18]. For instance, NF-κB promoted cancer cell proliferation via inducing the expression of chemokine receptor CXCR4 [19,20].

As the influence of PL on cell cycles has not been completely understood in the context of breast cancer cells, in the present study, the effects of PL on cell proliferation, cell cycles, and cell cycle-regulatory proteins were examined in MCF-7 cells. Furthermore, the involvement of ROS and the underlying intracellular signaling pathways associated with PL-induced cell cycle arrest was investigated. The outcome of this study may provide insights into the potential role of PL as a natural anticancer molecule.

## 2. Materials and Methods 

### 2.1. Materials

Dulbecco’s modified Eagle medium (DMEM), fetal bovine serum (FBS), penicillin/streptomycin antibiotic solution, and trypsin were obtained from Welgene (Gyeongsan, Korea). Phosphate-buffered saline (PBS) was purchased from Lonza (Basel, Switzerland). Piperlongumine was obtained from INDOFINE Chemical Company, Inc. (Hillsborough Township, NJ, USA). N-acetylcysteine (NAC), trypan blue solution, and 3-(4,5-Dimethylthiazol-2-yl)-2,5-diphenyltetrazolium bromide (MTT) were obtained from Amresco (Solon, OH). Dimethyl sulfoxide (DMSO) and 2′, 7′-dichlorofluorescin diacetate (DCFH-DA) were purchased from Sigma (St. Louis, MO, USA). An NF-κB inhibitor (Bay 11-7082), and antibodies against cyclin D1, CDK4, CDK6, proliferating cell nuclear antigen (PCNA), NF-κB p65, IκBα, lamin B, glyceraldehyde 3-phosphate dehydrogenase (GAPDH), goat anti-rabbit IgG-HRP, and donkey anti-goat IgG-HRP were purchased from Santa Cruz Biotechnology (Santa Cruz, CA, USA). Antibodies against cyclin B1, p-CDK1, and p-IκBα were obtained from Cell Signaling Technology (Danvers, MA, USA). IκB kinase-β antibody (IKK-β) was purchased from Abcam (Cambridge, MA, USA).

### 2.2. Cell Culture and Treatment

MCF-7 cells were obtained from KCLB (Korean cell line bank, Seoul, Korea). Cells were routinely cultured in DMEM supplemented with 10% FBS, 100 units/mL penicillin, and 100 μg/mL streptomycin at 37 °C in a humidified atmosphere containing 5% CO_2_. Cells were grown to approximately 50% confluency and then synchronized overnight in cell culture medium containing 1% FBS. Prior to treatments, the culture medium was replaced with a medium containing 10% FBS. Cells were pre-treated with 5 mM NAC for 1 h and were subsequently treated with PL (0, 5, 10, 20, and 40 µM) and the pharmacological inhibitor Bay 11-7082 (10 and 20 µM) at different time points depending on each experimental setting. 

### 2.3. Cell Proliferation Assay 

When cells reached approximately 40% to 50% confluency, cell proliferation was assessed using an MTT assay and the trypan blue dye exclusion test. To perform the trypan blue dye exclusion test, cells were seeded in 6-well plates and treated with PL (0, 5, 10, 20, and 40 µM) for 24 h. Cells were dissociated using trypsin, collected and centrifuged at 100× *g* for 5 min. The pellets were re-suspended in PBS. The number of viable cells was counted manually using a hemocytometer. To perform MTT assay, cells were seeded in a 96-well plate and were pre-treated with NAC (5 mM) for 1 h followed by treatment with PL (10 and 20 µM) or Bay 11-7082 (10 and 20 µM) for 24 h. Next, MTT reagent was added to each well followed by incubation for 3 h. Then, acidic isopropanol was added to each well to dissolve the deposited formazan. The optical density was determined at 570 nm on a spectrophotometer (Biotek Instrument, Winooski, VT, USA).

### 2.4. Wound Healing (Scratch) Assay

Cells were grown in 6-well plates up to 90% confluency and treated with PL (0, 5, 10, 20, and 40 μM). Wounds were made on the monolayer of cells using a sterile pipette tip, after that the cells were observed for 24 h. The wounds were photographed using a light microscope (40× magnification). To estimate the width of scratches, four different sites per scratch were observed.

### 2.5. Cell Cycle Analysis

Cell cycle distribution was analyzed as previously described [21]. Cells were treated with PL (0, 10, and 20 μM) for 24 h. Then, the cells were fixed and permeabilized with 70% cold ethanol at 4 °C for 16 h. After washing with PBS, the cells were resuspended in 500 μL of PBS, and then 50 μL of RNase A (Sigma, St. Louis, MO, USA) was added—so that a final concentration of 2 mg/mL was reached—and incubated at 37 °C for 2 h. The cells were then stained with 0.1 mg/mL propidium iodide (Sigma, St. Louis, MO, USA). Cell cycle distribution was measured using a CytoFLEX flow cytometer (Beckman Coulter, Indianapolis, IN, USA) and the data were analyzed by CytExpert software, version 2.0 (Beckman Coulter, Indianapolis, IN, USA).

### 2.6. Real-Time Polymerase Chain Reaction (PCR) Analysis 

Total RNA was extracted from the cells using TRIzol reagent (Ambion, Austin, TX, USA). Reverse transcription was performed using the TOPscript RT DryMIX kit (Enzynomics, Daejeon, Korea). mRNA expression was determined by real-time PCR using the Roche LightCycler^®^ 96 System (Roche, Basel, Switzerland) and 2× real-time PCR mix (SolGent, Daejeon, Korea). The PCR conditions were as follows: 95 °C for 15 min; 40 cycles of 95 °C for 20 s, and 58 °C for 40 s; 60 °C for 30 s; and a hold at 4 °C. Data were analyzed by the relative quantification method (ΔΔCq), using the house-keeping gene GAPDH as the internal control. The primer sequences are listed in Table 1.

### 2.7. Preparation of Cell Lysate and Western Blot Analysis

Cells were collected and lysed in RIPA-buffer and a protease inhibitor cocktail. Lysed cells were centrifuged at 21,000× *g* for 15 min at 4 °C. Protein concentration was measured using the Pierce BCA protein assay kit (Sigma-Aldrich, St. Louis, MO, USA) and cell lysates were stored at −80 °C until further use. For Western blot, protein samples (30 µg per treatment) were separated by sodium dodecyl sulphate-polyacrylamide gel electrophoresis (SDS-PAGE) and transferred to nitrocellulose membranes. Following protein transfer, membranes were blocked with 3% non-fat milk buffer and then incubated overnight at 4 °C with primary antibodies, which were used at a dilution range of 1:1000 to 1:20,000. After washing, the membranes were incubated with horseradish peroxidase (HRP)-conjugated secondary antibodies (1:5000). The membranes were visualized using enhanced chemiluminescence (ECL) reagents (Thermo Fisher Scientific, Waltham, MA, USA). The density of the bands was determined using Image J software (National Institutes of Health, Bethesda, MD, USA), and normalized to that of the house-keeping protein, GAPDH.

### 2.8. Measurement of Reactive Oxygen Species Generation

MCF-7 cells were grown to confluence in 6-well plates. Cells were pre-treated with or without 5 mM NAC for 1 h followed by PL treatment (0, 5, 10, and 20 µM) for 3 h. Following the treatments, cells were incubated with 2’,7’-dichlorofluorescin diacetate (DCFH-DA) (final concentration, 20 μM) at 37 °C in a 5% CO_2_ incubator for 30 min. Cells were washed 3× with PBS to terminate the reaction. The generation of H_2_O_2_ was evaluated using an Olympus IX71 fluorescence microscope (Olympus Optical Co. Ltd., Tokyo, Japan) and the fluorescent images were captured using an Olympus DP71 camera and DP controller software, version 2.2 (Olympus Optical Co. Ltd., Tokyo, Japan).

### 2.9. Measurement of Glutathione Level

Intracellular glutathione (GSH) level was measured using a commercial assay kit (BioVision, Mountain View, CA, USA). Briefly, control and treated cells (1 × 10^6^) were collected by centrifugation at 700× *g* for 5 min and lysed in cell lysis buffer at 4 °C for 10 min. Then, they were centrifugated again at 21,000× *g* for 10 min and the supernatants were transferred into a new tube for the assay and were then pipetted into a 96-well plate. Next, the glutathione S-transferase (GST) reagent and monochlorobimane were added to each sample in the 96-well plate and then the plate was incubated at 37 °C for 1 h. The fluorescence was measured by a fluorescence microplate reader (Molecular Devices, San Jose, CA, USA) at 360/460 nm (excitation/emission wavelength).

### 2.10. Nuclear Fractionation

Cells were seeded in a 10-cm plate and treated with PL (20 μM, 0–24 h). Cells were lysed in a hypotonic buffer solution (20 mM Tris (pH 7.4), 10 mM NaCl, 3 mM MgCl_2_, 0.5 mM PMSF, and 1 mM NaF) containing a protease inhibitor cocktail. After the addition of 10% Triton-X 100, cell lysates were centrifuged at 12,000× *g* for 5 min at 4 °C. The supernatants were the cytosolic fraction. Pellets were resuspended in cell extraction buffer (100 mM Tris (pH 7.4), 2 mM NaOV, 100 mM NaCl, 1% Triton X-100, 1 mM EDTA, 10% glycerol, 1 mM EGTA, 10% SDS, 0.5 mM PMSF, 1 mM NaF, and 20 mM Na_4_P_2_O_7_) and a protease inhibitor cocktail. The homogenates were centrifuged at 12,000× *g* for 10 min at 4 °C, and the supernatants (nuclear fraction) were used to determine nuclear translocation of NF-κB.

### 2.11. Statistical Analysis

Data were expressed as mean ± standard error of the mean (SEM). Statistical significance was determined with SPSS-PASW statistical software, version 18.0 for Windows (SPSS, Chicago, IL, USA) using independent two-sample *t*-test, and one-way ANOVA and the differences between groups were compared using Tukey’s post hoc test. A *p* value of <0.05 was considered statistically significant.

## 3. Results

### 3.1. PL Suppresses Cell Proliferation and Migration

**Figure 1:**
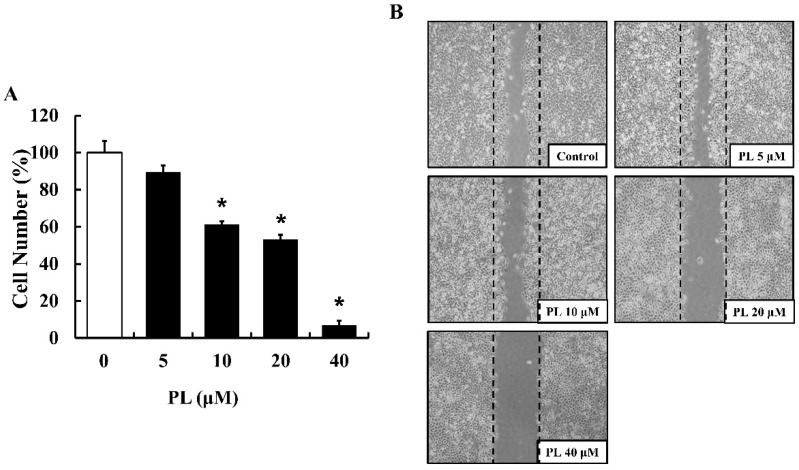
Effects of piperlongumine (PL) on the proliferation and migration of MCF-7 cells. (**A**) Cell viability was determined using the trypan blue dye exclusion test. Data represent the mean ± SEM (n = 3). * Significantly different compared with the control (*p* < 0.05). (**B**) Cell migration was evaluated using a wound healing assay. The widths of the wounds were photographed using an inverted microscope (40× magnification). The images shown are representatives of three independent experiments.

To determine the effect of PL on the proliferation and migration of human breast cancer cell line MCF-7, the trypan blue dye exclusion test and a wound healing assay were performed, respectively. As shown in Figure 1A, PL inhibited the proliferation of MCF-7 cells in a dose-dependent manner, and a significant decrease in cell number was observed at a concentration range of 10–40 µM PL, compared to that in the control. In addition, the wound healing assay revealed that PL inhibited MCF-7 cell migration in a dose-dependent manner (Figure 1B). 

### 3.2. PL Induces Cell Cycle Arrest in MCF-7 Cells

**Figure 2:**
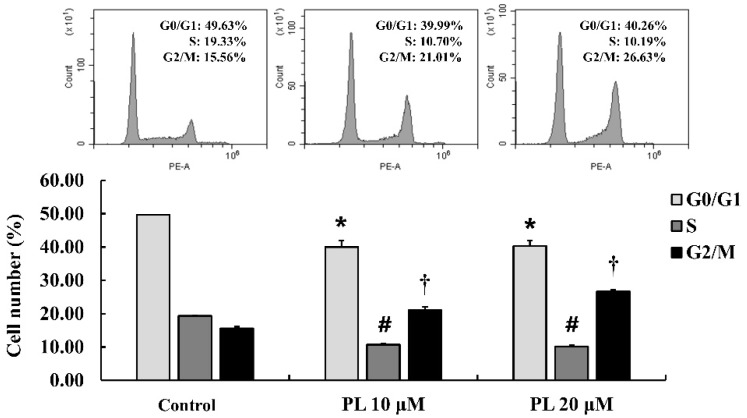
Effects of piperlongumine (PL) on cell cycle distribution in MCF-7 cells. Cell cycle distribution was determined using flow cytometry and analyzed with CytExpert software. Data represent the mean ± SEM (n = 3). *^,#,†^ Significantly different compared with that of the control (*p* < 0.05).

To investigate the effect of PL on the cell cycle, a flow cytometry assay was performed to measure cell cycle distribution. The results showed that the distribution of cells in G2/M phase was significantly increased by 5.56% (10 µM PL) and 11.07% (20 µM PL), compared to that in the control (Figure 2). These results demonstrated that PL suppressed cell proliferation through G2/M arrest during the cell cycle.

### 3.3. PL Modulates Cell Cycle-Regulatory Proteins

**Figure 3:**
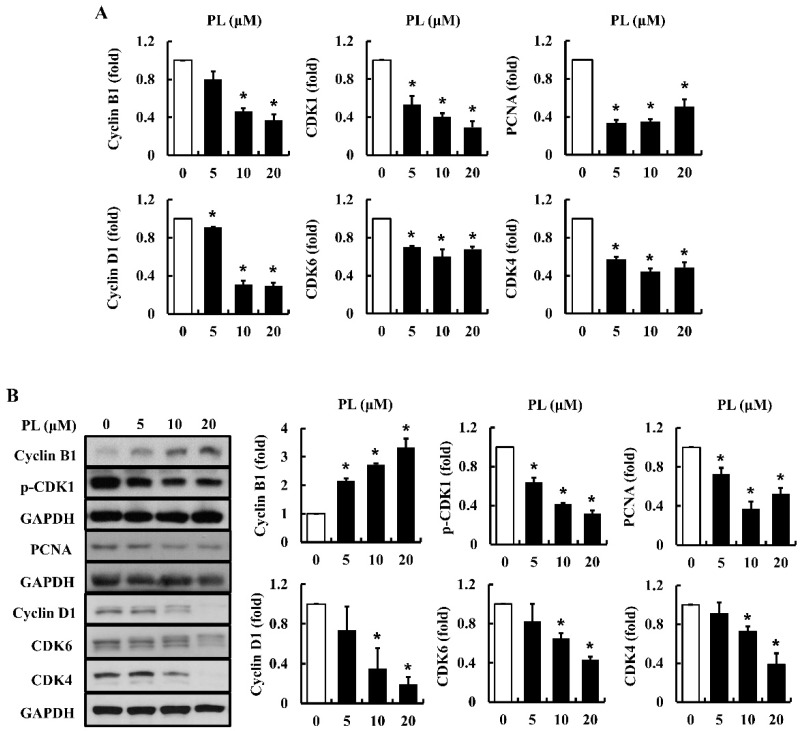
Effects of piperlongumine (PL) on cell cycle-regulatory proteins in MCF-7 cells. The expression of cyclin B1, p-CDK1, CDK1, PCNA, cyclin D1, CDK6, and CDK4 at mRNA (**A**) and protein levels (**B**) was measured using real time PCR and Western blotting, respectively. GAPDH was used as an internal control gene and as a loading control. Data represent the mean ± SEM (n = 3). * Significantly different compared with the control (*p* < 0.05).

The proliferation of cancer cells is regulated by cell cycle-related molecules, such as cyclins and CDKs [22]. To examine the effects of PL on the mRNA and protein expression of cell cycle-associated molecules in MCF-7 cells, real-time PCR and Western blot analysis were performed. The results showed that the expression of cyclin B1, cyclin D1, CDK1, CDK4, CDK6, and PCNA mRNA was significantly decreased in cells treated with PL, especially at 10 and 20 µM concentrations (Figure 3A). In addition, the expression of cyclin D1, p-CDK1, CDK4, CDK6, and PCNA protein was decreased in cells treated with PL (Figure 3B). PL increased the expression of cyclin B1 at the protein level, while decreasing it at the mRNA level (Figure 3B). Our results suggest that PL arrests cell cycles through the modulation of mRNA and protein expression of cell cycle-related proteins (i.e., CDK1, CDK4, CDK6, cyclin B1, cyclin D1, and PCNA).

### 3.4. PL Induces Intracellular ROS Accumulation and GSH Depletion

**Figure 4:**
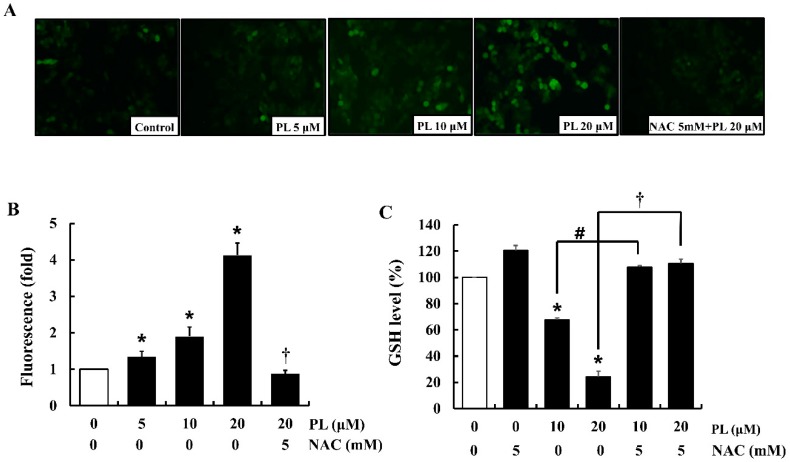
Effects of piperlongumine (PL) on ROS level and intracellular GSH in MCF-7 cells. (**A**) Cells were stained with DCFH-DA to detect ROS (H_2_O_2_) production. The intensity of green fluorescence (H_2_O_2_ production) was determined using a fluorescence microscope. The images shown are representatives of three independent experiments. (**B**) DCF-DA positive (green fluorescence) area was analyzed with Image J. (**C**) GSH levels were measured using a fluorometric assay. The fluorescence value was determined by a fluorescence microplate reader. Data represent the mean ± SEM (n = 3). * Significantly different compared with that of the control (*p* < 0.05). ^#^ Significantly different compared to that of 10 µM PL (*p* < 0.05). ^†^ Significantly different compared to that of 20 µM PL (*p* < 0.05).

Excessive production of intracellular ROS can modulate cell proliferation by regulating cell cycle-related proteins [23]. Therefore, in the present study, intracellular ROS (e.g., H_2_O_2_) levels were measured using DCFH-DA fluorescent dye which emits green fluorescence in the presence of H_2_O_2_. The results showed that PL significantly increased H_2_O_2_ levels (green fluorescence) in a dose-dependent manner, while both the negative control and NAC-treated cells attenuated PL-induced intracellular production of H_2_O_2_ (Figure 4A,B). Furthermore, to determine the cellular events related to PL-induced ROS accumulation and oxidative stress, cellular GSH levels were measured. The results showed that PL significantly decreased cellular GSH levels, while in cells pre-treated with NAC, the GSH levels were similar to those observed in control cells (Figure 4C). 

### 3.5. PL Decreases Nuclear Translocation of NF-κB p65

**Figure 5:**
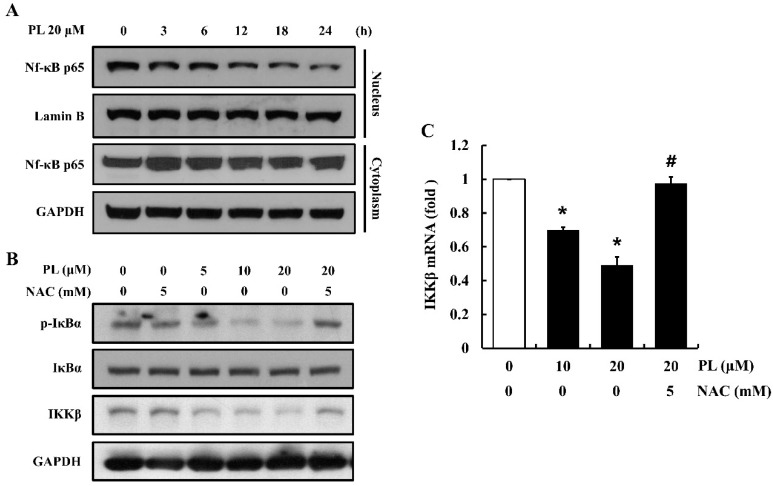
Effects of piperlongumine (PL) on NF-κB activation in MCF-7 cells. (**A**) The nuclear translocation of Nf-κB p65 was determined using nuclear fractionation and Western blotting. Lamin B and GAPDH were used as loading controls. (**B**) The expression of p-IκBα and IKKβ was examined by Western blotting. GAPDH and IκBα were used as loading controls. (**C**) The mRNA expression of IKKβ was determined using real time PCR. GAPDH was used as the internal control gene. The images shown are representatives of three independent experiments. Data represent the mean ± SEM (n = 3). * Significantly different compared with the control (*p* < 0.05). ^#^ Significantly different compared to 20 µM PL (*p* < 0.05).

The NF-κB pathway has been known to play a critical role in cell proliferation [24]. To evaluate whether PL has an influence on the activation of NF-κB in cells, nuclear fractionation and Western blotting were performed. PL attenuated the nuclear translocation of NF-κB p65 by decreasing IκBα phosphorylation, as well as the expression of IKKβ at mRNA and protein level (Figure 5A–C). In addition, pre-treatment of cells with NAC prevented PL-induced decrease in IκBα phosphorylation and IKKβ gene expression (Figure 5B,C). These data suggest that PL attenuates the activation of the NF-κB pathway via decreasing IKKβ expression caused due to ROS accumulation.

### 3.6. PL Increases the Expression of p21 mRNA

**Figure 6:**
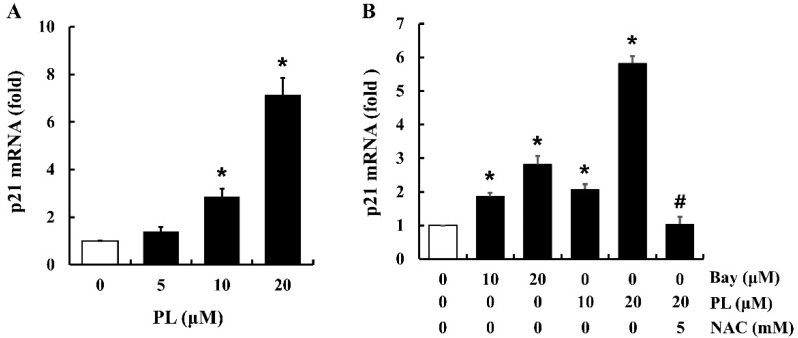
Effects of piperlongumine (PL) on p21 mRNA expression in MCF-7 cells. The mRNA level of p21 in PL-treated cells was measured using real time PCR. (**A**) Cells were treated with PL (0, 10, and 20 µM) for 12 h. (**B**) Cells were treated with Bay 11-7082 (Bay; NF-κB inhibitor; 10 and 20 µM) or PL (10 and 20 µM) for 12 h, with or without NAC pretreatment (5 mM, 1 h). GAPDH was used as the internal control gene. Data represent the mean ± SEM (n = 3). * Significantly different compared with the control (*p* < 0.05). ^#^ Significantly different compared to 20 µM of PL (*p* < 0.05).

According to previous studies, the p21 protein can attenuate the activation of CDKs (i.e., CDK1, CDK4, and CDK6) and the PCNA protein [25]. To identify the cellular signaling pathways associated with PL-induced cell cycle arrest, the expression of p21 mRNA was determined in cells treated with PL. Treatment of cells with PL increased p21 mRNA expression in a dose-dependent manner (Figure 6A). Furthermore, to investigate the correlation between PL-induced ROS accumulation, NF-κB inactivation, and the increase in p21 mRNA expression, cells were pre-treated with NAC (a ROS scavenger) for 1 h followed by treatment with PL or Bay 11-7082 (an NF-κB inhibitor) for 12 h. Both PL and Bay 11-7082 increased the expression of p21 mRNA (Figure 6B). NAC significantly inhibited PL-induced overexpression of p21 mRNA, compared to that of 20 µM of PL alone (Figure 6B). These results indicate that PL increases the expression of p21 mRNA by suppressing NF-κB activation through ROS accumulation.

### 3.7. PL Suppresses Cell Proliferation by Accumulating ROS and Inactivating NF-κB

**Figure 7:**
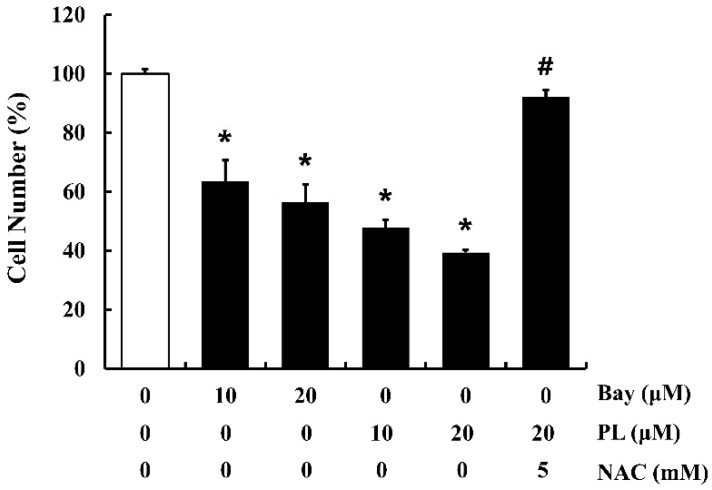
Effects of Bay 11-7082 (Bay; NF-κB inhibitor), piperlongumine (PL), and NAC on proliferation of MCF-7 cells. Cells were treated with Bay 11-7082 (10 and 20 µM) or PL (10 and 20 µM) for 24 h, with or without NAC pretreatment (5 mM, 1 h). An MTT assay was performed to determine the number of viable cells. Data represent the mean ± SEM (n = 4). * Significantly different compared with the control (*p* < 0.05). ^#^ Significantly different compared to 20 µM of PL (*p* < 0.05).

To confirm the underlying mechanism of the anti-proliferative effect of PL, cells (approximately 40–50% confluency) were pre-treated with NAC for 1 h, followed by treatment with PL or Bay 11-7082 for 24 h. PL and Bay 11-7082 significantly suppressed cell proliferation, while pretreatment of cells with NAC reversed PL-induced anti-proliferation effects (Figure 7). These results demonstrate that both ROS and NF-κB might play critical roles in PL-induced anti-proliferation effects in MCF-7 cells.

## 4. Discussion

Approximately 70% of breast cancer cases are ER-positive [26]. Generally, patients with ER-positive breast cancer are treated with endocrine therapy, which targets either the activity of ER with selective ER modulators or the production of estrogen with aromatase inhibitors [27]. Unfortunately, resistance against endocrine therapy is frequent in early and metastatic breast cancer, resulting in treatment failure and relapse [28,29]. Therefore, there is a need to develop effective treatment strategies for ER-positive breast cancer that compensates conventional endocrine therapy. 

PL has been known to be an anticancer phytochemical, and previous studies in various cancer cells, such as malignant melanoma cells, colorectal cancer cells, and lung cancer cells, have demonstrated that the anticancer effects of PL are mainly related to apoptosis [21,30,31]. Other studies have found that PL also exerts its anticancer effects via inhibition of the STAT3 and Akt/mTOR signaling pathways in breast cancer cell lines [11,32]. As mentioned above, there are several reports regarding the anticancer effects of PL, but studies related to specific cell cycle checkpoint proteins are very few. Thus, in our study, PL-induced anti-proliferative effects associated with the expression of cell cycle checkpoint proteins, and the underlying cellular mechanisms, were examined in an ER-positive breast cancer cell line, MCF-7.

PL inhibited cell proliferation and migration in a dose-dependent manner (10–40 μM). As cell cycle progression mechanically controls cell proliferation, it is closely associated with cancer development, which is induced by abnormal cell proliferation [33]. The cell cycle includes four phases, G1, S, G2, and M phase, and there are important checkpoints to regulate cell cycle progression. Thus, based on the anti-proliferative effect of PL, cell cycle distribution was examined using flow cytometry. In subsequent experiments, the expression of cell cycle-regulatory molecules at mRNA and protein levels was observed at various cell cycle checkpoints. Cell cycle progression was significantly arrested at the G2/M phase in cells treated with PL. Moreover, PL inhibited the expression of all checkpoint proteins at mRNA and protein levels, with the exception of cyclin B1 (Figure 3). In the cell cycle progression, a decrease of cyclin B1 via degradation is an essential step to exit from the M phase and progress into the next cell division [34]. In the present study, the mRNA level of cyclin B1 was decreased (Figure 3A). However, the protein expression of cyclin B1 was increased by PL in a dose-dependent manner (Figure 3B). It seems PL inhibited the degradation of cyclin B1 and that led to cell cycle arrest at the G2/M phase. A previous study has also demonstrated that PS-341, a boronic acid dipeptide, induced G2/M phase arrest through a blockade of cyclin B1 degradation in human non-small cell lung cancer cells [35].

Excessive generation of cellular ROS is known to be the major mechanism through which PL exerts its anticancer effects [36,37]. Our data also showed that PL increased ROS accumulation in MCF-7 cells, while pretreatment of cells with NAC attenuated this accumulation. Pretreatment of NAC to cells might be involved in scavenging ROS and restoring GSH in cells. In fact, NAC possesses a thiol group that scavenges ROS and is also a precursor of cellular GSH [38,39]. In agreement with our data, a previous study reported that PL induces ROS accumulation and GSH depletion in glioblastoma multiforme cells [40]. In fact, cancer cells have a higher level of endogenous ROS than normal cells, which is associated with the abnormal growth of cancer cells [41]. Particularly, mitochondria and nicotinamide adenine dinucleotide phosphate oxidases are known as main contributors of increased ROS in cancer cells [42]. The elevated ROS level in cancer cells triggers the increase in antioxidant capacity and eventually enables the balance of the cellular redox system [43]. This adaptation to ROS stress is a necessary step for cancer cells to survive. Our data demonstrated that PL-induced ROS exceeded the antioxidant capacity of MCF-7 cells that led to the disruption of the cellular redox balance. Damages of cellular redox system can result in severe cell damage including the inhibition of cell proliferation, abnormal signaling, accumulation of harmful by-products, and apoptosis [44,45]. Furthermore, according to previous studies, ROS affected on cell cycle progression via modulating cell cycle regulators, such as cyclins, cyclin dependent kinase inhibitor, and Cdc25 [46,47]. Particularly, H_2_O_2_ induced multi-phase cell cycle arrest via a decrease of cyclins D expression and an increase of p21 expression [46]. These past data and our results suggest that ROS regulates multiple cell cycle regulators including p21, cyclins, and CDKs. 

To identify the underlying mechanisms of PL-induced cell cycle arrest via ROS accumulation, the IKK/NF-κB activation pathway was investigated using Western blotting and the nuclear fractionation technique. The IKK/NF-κB pathway has been known to play a key role in the proliferation and metastasis of cancer cells [48]. It was previously been shown that PL decreased the proliferation of lung cancer cells through reducing the nuclear translocation of NF-κB [21]. In accordance with the previous data, our results also showed that PL attenuated the activation of the IKK/NF-κB pathway in breast cancer cells. Further, our study revealed that this deactivation of NF-κB occurred through decreased IKKβ expression due to the accumulation of PL-induced ROS, which is known to target IKK and induce IKKβ S-glutathionylation, resulting in the inhibition of NF-κB activation [17]. Moreover, cells treated with PL showed increased expression of p21 mRNA, which is known to play a key role in the G2/M phase arrest through negatively regulating CDK1 activation [49]. According to previous studies, NF-κB deactivation decreased mouse double minute 2 homolog (MDM2) expression, which is a significant negative regulator of p53, leading to cell cycle arrest along with an increased p21 expression [50,51].

## 5. Conclusions

PL attenuated MCF-7 cell proliferation by suppressing IKKβ expression through the accumulation of ROS and resulting cellular oxidative stress. Our study also provides in vitro evidence that PL could serve as a potential anticancer molecule that compensates conventional endocrine therapy in ER-positive breast cancer.

## Figures and Tables

**Table 1:** Primers used for real-time PCR.

Genes	Primer Sequences (5′–3′)	Accession Numbers
Cyclin B1	(F) GTA AGC CAA GTC ATG GAG AAT C (R) GCA GCA ATC ACA AGA AGA AAC	BC006510.2
CDK1	(F) TGT CCG CAA CAG GGA AGA AC (R) CGA AAG CCA AGA TAA GCA ACT C	BC107750.1
PCNA	(F) AAA TGC GCC GGC AAT GAA GA (R) TTC CTG TAG CTT CGT GAC TCG GTA	BC062439.1
Cyclin D1	(F) CCC CGC ACG ATT TCA TTG AAC A (R) TGG AGG GCG GAT TGG AAA TGA A	NM_053056.3
CDK6	(F) GAT GTT TCA GCT TCT CCG AGG TCT (R) AAG GCC GAA GTC AGC GAG TTT T	BC052264.1
CDK4	(F) GGT AAC CCT GGT GTT TGA GCA TGT (R) GCG CAT CAG ATC CTT GAT CGT TTC	NM_000075.4
IKKβ	(F) AAA ACC TCG AGA CCA GCG AAC T (R) GCT GCG TAT AGA TCA CTC GCA CTT	AF080158
p21	(F) TGG AGA CTC TCA GGG TCG AAA (R) GGC GTT TGG AGT GGT AGA AAT C	BC000312.2
GAPDH	(F) GAC CCC TTC ATT GAC CTC AAC TAC (R) ATG ACA AGC TTC CCG TTC TCA G	DQ403057.1
